# Supplementary material for: Dual Effect of Chemo-PDT with Tumor Targeting Nanoparticles Containing iRGD Peptide
Source: Pharmaceutics. 2023 Feb 11;15(2):614. doi: 10.3390/pharmaceutics15020614 (PMC9959063; doi:10.3390/pharmaceutics15020614)
Supplement: Supplementary file 1 [file pharmaceutics-15-00614-s001.zip › pharmaceutics-2129587-supplementary.pdf]

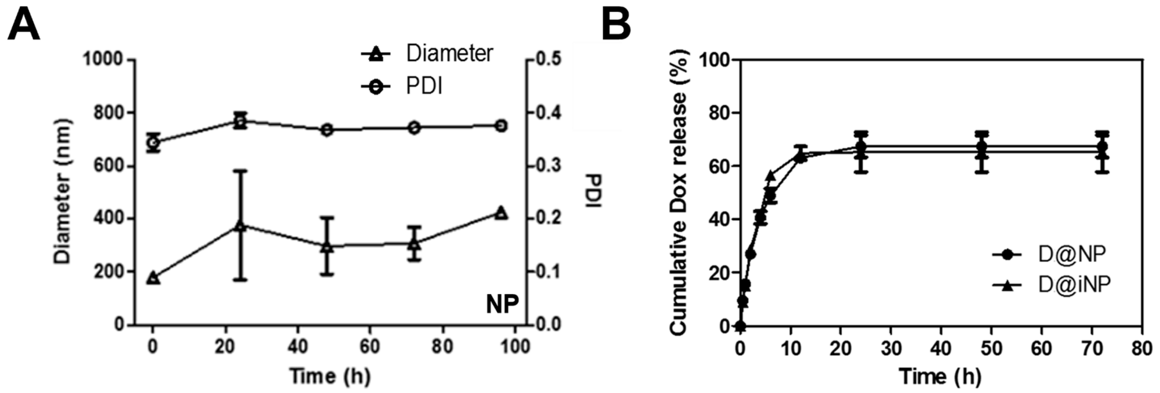

Figure S1: Characterization of nanoparticles. (A) Serum stability of NP for 96 h. (B) Dox release from D@NP and D@iNP in PBS at 37 °C.

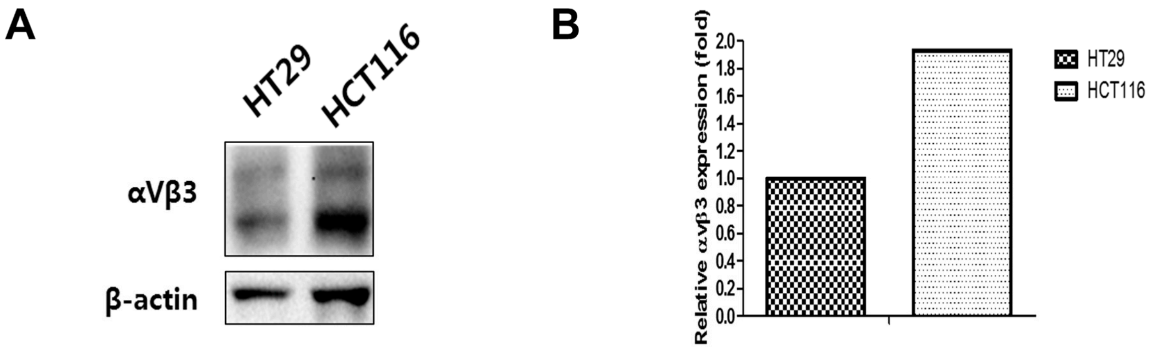

Figure S2: Detection of $\alpha v\beta 3$ and cellular uptake in HT29 and HCT116. (A) Western blot of integrin $\alpha v\beta 3$ in HT29 and HCT116. (B) Quantitative analysis of Western blot

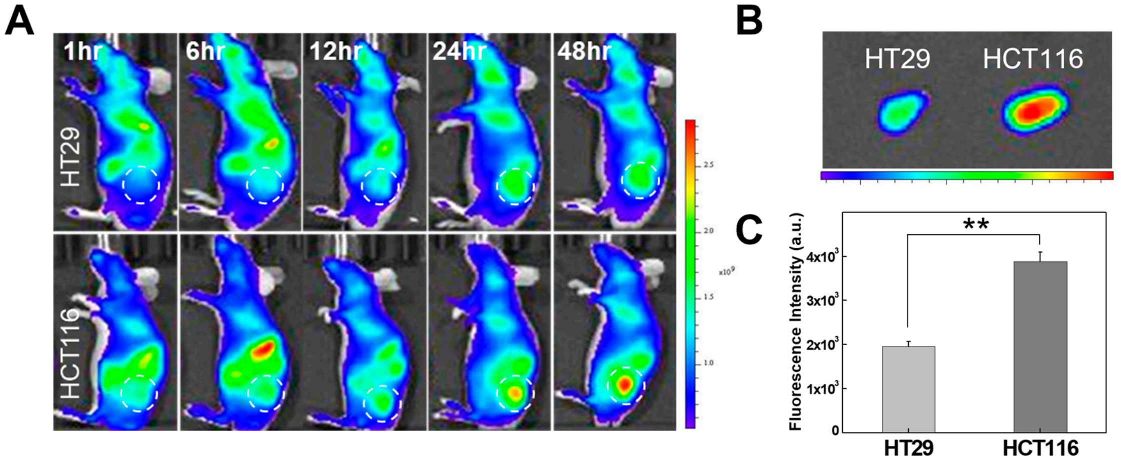

Figure S3: Biodistribution of D@iNP in HCT116 and HT29 tumor-bearing mice. Fluorescence signals were monitored in NIRF channel based on the intrinsic fluorescence of Ce6 in D@iNP. (A) Whole body fluorescence images of mice after intravenous injection of D@iNP. (B) Ex vivo NIRF imaging of the tumor extracted 48 h post injection of D@iNP. (C) NIRF intensities of tumor tissues in (B). ** indicates differences at the $p < 0.01$ significance levels (analyzed using one-way ANOVA).
